# Fighting a Universal Plague

**Published:** 1921-06-04

**Authors:** 


### THE PUBLIC WELL-BEING.

### Fighting a Universal Plague.

### THE NEED FOR COMMON ACTION.

In view of the unsatisfactory differences of opinion which still prevail with regard to the methods of protecting the public against venereal disease—differences very strikingly exemplified in the controversy which has been proceeding for many weeks in the columns of the *New Statesman*—it is surely time that some authoritative attempt should be made to unify and direct public opinion on this grave question, and to establish a common basis of action. The pressing importance of the problem in its national aspects was never more clearly or openly recognised than it is to-day, but there is a very real danger lest all the moral energy which has been generated should be frittered away in futile disagreements as to the policy to be pursued. We wish, on that account alone, that a wider publicity could be given by means of a summarised translation (by some expert in the Ministry of Health) of the notable book recently published by Dr. Arthur Vernes, director of the Institut Prophylactique of Paris, in which he plainly faces the facts of the situation, and goes straight to the point in considering the methods to be employed in combating the disease.

Presenting his view, as affirmed in his own publication ("Atlas de Syphilimétrie, les conditions expérimentales de l'exti[illegible]tion de la syphilis"), to the League of Red Cross Societies, he points out that of all diseases spoken of as social, because they prey upon the society which harbours them, syphilis is the most common. At the head of 25,633 cases of infectious diseases notified in New York during the period July 4 to October 3, 1914, comes syphilis, with 6,432 cases, or 25 per cent. Tuberculosis follows with 5,525 cases, or 21 per cent., then come diphtheria, measles, scarlet fever, etc. These figures are the more striking because syphilis is of all diseases the most often unrecognised or concealed, and that in which unnotified cases are the most numerous. This proportion is by no means peculiar to the United States. According to Dr. Vernes' personal inquiries, it would seem that, at the present time, in Paris and its neighbourhood, the proportion of syphilitics in the population may be estimated at one-quarter. In some other countries it is generally endemic.

Of all social diseases syphilis is likewise the most deadly and most insidious, since it strikes not only the living, but the unborn. Its toxin permeates to the very germ of life, changing and destroying it. Ubiquitous, it is yet invisible, and therein lies the source of its very formidable strength. It is possible to be unaware of syphilis at the time when it is transmissible, and then to be fatally injured in heart or in brain without suspecting the cause. Everywhere in process of extension, syphilis represents in society what the silent uprising of a submarine peak on a great sea route would represent to the navigator. For the future of the race it is a terrible danger, often unrecognised even by those most actively interested in social hygiene. Dr. Vernes speaks of a well-known philanthropist "who has consecrated his energies with regal generosity to the subject of repopulation," and yet was able recently to declare that the fight against syphilis had no place in his programme!

It is certainly true that, in nearly all civilised countries, the cry of alarm has been raised and steps have been taken to stem the plague. One can only prophesy success for these attempts if they are organised according to a uniform plan adapted to the end in sight, but, on the contrary, they follow very different lines of action, and this very diversity "delays the general uncertainty in the combat against syphilis, an uncertainty which, in default of a directing idea, results in a great variety of campaigns, each following the prevalent customs and opinion." In Great Britain, the country of individual freedom, says one author, patients obtain treatment where and how they will, without any official direction (he is not quite up to date in this remark), whilst in Germany, the country of disciplined organisation, consultation ports, spread all over the land, constitute a veritable network of venereal surveillance and pass on their patients either to private consulting rooms or to public institutions, all at the expense of insurance societies which are interested in seeing that their clients with venereal disease shall be watched and treated as soon as their condition demands it. In Switzerland every town arranges its anti-venereal organisations as it thinks best, basing its action on the federal law concerning epidemic and contagious diseases. Scandinavia strives uniformly to dry up the springs of evil by applying to the germ of syphilis the same general rules that are applied to any virus of contagion, that is to say, the rules of discovery and disinfection.

This legislation, while apparently logical, offers, in the view of Dr. Vernes, serious inconvenience—violation of professional secrecy, intrusion into private life, denunciations—only to end with a fatally incomplete result. In the United States the same principle of staying and preventing the diffusion of infection is pushed much further still, to the point of arresting those suspected of infection, to the quarantining of infected persons, and even to the inclusion of illicit sexual relations among the punishable offences. Dr. Vernes does not seek apparently to ignore the significance of efforts towards morality rightly conceived and nobly practised, especially in Switzerland and in the United States, through education, writings, books, posters, and cinematograph films. But these means of action, which aim at a transformation of habit, can only save the race a long time from now, and the pressing necessity which permits of no delay is to face the danger of the extension of syphilis and to encompass the extinction of the infectious germs wherever they are to be found. About ten years ago he conceived

**THE PUBLIC WELL-BEING—(*continued*).**

the possibility of giving a scientific basis to the fight against syphilis by realising what the master of French syphiligraphy, Professor Fournier, has called " prophylaxis by treatment."

When the Institut Prophylactique was founded in Paris in 1910 he asserted that it was possible to put at the disposition of the public a method of observing syphilitic infections which would permit observation step by step of the effects of treatment, and that then it would be possible to attain arrest of infection and the suppression of hereditary syphilis. He now claims that the documents which he reproduces show that the employment of anti-syphilitic remedies may be checked by a control which increases tenfold the effect and gives results with a certainty undreamed of before, and that the popularisation of these procedures would make possible the arrest, and, finally, the extinction of syphilis. This is a high claim which must be put very severely to the test before it can generally be accepted, but Paris has certainly set an example to this country of enterprise and research in the right direction. The Institut is a demonstration of what can be done at the present time quietly and with little cost. Unsuspected syphilis is unmasked and the precise extent to which a patient remains infected after a given treatment can be defined. Moreover, the Institut appears to be a spontaneous focus of publicity, by sparing patients pain, saving their time, and inducing their confidence to such an extent that they invariably recruit other patients.

The protection of the public against the universal plague of venereal disease requires an immense and widespread effort. It is evident that prophylaxis by treatment should be organised on solid scientific foundations. But in England the public is not yet awakened to the necessity for a great intelligently directed onslaught on the disease. The Government is fully aware of the danger and of the necessity for action, but its will and its concentration of purpose seem to be weakened by the warring antipathies of various organisations and authorities that concern themselves with the subject.

